# Necessity of our successful mitral valve repair in a severe mitral regurgitation case with many co-morbid factors

**DOI:** 10.1186/1749-8090-8-S1-P127

**Published:** 2013-09-11

**Authors:** A Gurbuz, U Yetkin, S Yazman, N Karakaş, I Yurekli, N Postaci, C Nazli

**Affiliations:** 1 Department of Cardiovascular Surgery, Izmir Katip Celebi University Ataturk Training and Research Hospital, Izmir, Turkey; 2 Department of Cardiology, Izmir Katip Celebi University Ataturk Training and Research Hospital, Izmir, Turkey

## Background

The superiority of mitral valve repair over replacement is widely accepted. Developing experience and scientific improvement made mitral valve repair techniques more prominent particularly in degenerative valvular disorders.

## Methods

Our case was a 62-year-old male. He was suffering from shortness of breath and easy fatigability for 5 years. His past medical history was significant for upper gastrointestinal bleeding three times in the past 20 years. Transthoracic echocardiography revealed severe mitral regurgitation. Transesophageal echocardiography (TEE) confirmed severe mitral regurgitation caused by severe prolapsus of both leaflets.

## Results

He was taken into operating room with these findings. After left atriotomy, saline test showed severe mitral regurgitation caused by P3 scallop of posterior leaflet particularly. A quadrangular resection was made on P3 scallop. Then, mitral ring annuloplasty was performed using SJM Tailor Annuloplasty Ring (TARP-31). Saline test showed a minimal central regurgitation with complete coaptation and competence of both leaflets of native valve. After closure of left atriotomy at the time of decannulation, perioperative control TEE confirmed optimal native mitral valve coaptation. In this way, we avoided oral anticoagulant therapy for this patient with a past medical history of gastrointestinal bleeding. Late period outpatient follow-up controls are event-free.

## Conclusions

The key points in repair of mitral valve are planning of the repair technique after evaluation of the valvular pathology in detail preoperatively and experience of the surgeon. The excellence of the repair may be confirmed by the surgeon with naked eye directly or by perioperative TEE as in our case. Patient should not leave the operating room without the possibly best repair of the valve.

